# IGF1-Stimulated Posttraumatic Hippocampal Remodeling Is Not Dependent on mTOR

**DOI:** 10.3389/fcell.2021.663456

**Published:** 2021-05-20

**Authors:** Erica L. Littlejohn, Anthony J. DeSana, Hannah C. Williams, Rudy T. Chapman, Binoy Joseph, Jelena A. Juras, Kathryn E. Saatman

**Affiliations:** Department of Physiology, Spinal Cord and Brain Injury Research Center, University of Kentucky, Lexington, KY, United States

**Keywords:** traumatic brain injury, insulin-like growth factor-1, mTOR, neurogenesis, rapamycin, neuronal differentiation, pS6, dendritic outgrowth

## Abstract

Adult hippocampal neurogenesis is stimulated acutely following traumatic brain injury (TBI). However, many hippocampal neurons born after injury develop abnormally and the number that survive long-term is debated. In experimental TBI, insulin-like growth factor-1 (IGF1) promotes hippocampal neuronal differentiation, improves immature neuron dendritic arbor morphology, increases long-term survival of neurons born after TBI, and improves cognitive function. One potential downstream mediator of the neurogenic effects of IGF1 is mammalian target of rapamycin (mTOR), which regulates proliferation as well as axonal and dendritic growth in the CNS. Excessive mTOR activation is posited to contribute to aberrant plasticity related to posttraumatic epilepsy, spurring preclinical studies of mTOR inhibitors as therapeutics for TBI. The degree to which pro-neurogenic effects of IGF1 depend upon upregulation of mTOR activity is currently unknown. Using immunostaining for phosphorylated ribosomal protein S6, a commonly used surrogate for mTOR activation, we show that controlled cortical impact TBI triggers mTOR activation in the dentate gyrus in a time-, region-, and injury severity-dependent manner. Posttraumatic mTOR activation in the granule cell layer (GCL) and dentate hilus was amplified in mice with conditional overexpression of IGF1. In contrast, delayed astrocytic activation of mTOR signaling within the dentate gyrus molecular layer, closely associated with proliferation, was not affected by IGF1 overexpression. To determine whether mTOR activation is necessary for IGF1-mediated stimulation of posttraumatic hippocampal neurogenesis, wildtype and IGF1 transgenic mice received the mTOR inhibitor rapamycin daily beginning at 3 days after TBI, following pulse labeling with bromodeoxyuridine. Compared to wildtype mice, IGF1 overexpressing mice exhibited increased posttraumatic neurogenesis, with a higher density of posttrauma-born GCL neurons at 10 days after injury. Inhibition of mTOR did not abrogate IGF1-stimulated enhancement of posttraumatic neurogenesis. Rather, rapamycin treatment in IGF1 transgenic mice, but not in WT mice, increased numbers of cells labeled with BrdU at 3 days after injury that survived to 10 days, and enhanced the proportion of posttrauma-born cells that differentiated into neurons. Because beneficial effects of IGF1 on hippocampal neurogenesis were maintained or even enhanced with delayed inhibition of mTOR, combination therapy approaches may hold promise for TBI.

## Introduction

Annually, millions of people are living with long-term disability caused by traumatic brain injuries (TBI) (Taylor et al., 2017). The disruption of neural networks resulting from axonal and dendritic degeneration, neuronal cell death and a limited ability to replace and repair lost cells contributes to cognitive disability. The brain can respond to injury by stimulating forms of adaptive plasticity such as cellular reorganization, neural cell proliferation, angiogenesis, altered neurotransmitter release, and neurogenesis (Bramlett and Dietrich, 2015; McGinn and Povlishock, 2015). In particular, hippocampal neurogenesis may play an important role in cognitive recovery following trauma. Ablating neurons born after TBI impairs cognitive recovery (Blaiss et al., 2011). This is consistent with work directly linking enhanced neurogenesis to improvements in spatial learning, reference memory and pattern separation (Sahay et al., 2011; Burghardt et al., 2012; Bekinschtein et al., 2014). However, enhanced neurogenesis has also been associated with histological hallmarks of epilepsy (Parent et al., 1997; Jessberger et al., 2005; Butler et al., 2015), raising concern about therapies designed to drive increased hippocampal neurogenesis after brain injury. A better understanding of mechanisms underlying posttraumatic neurogenesis is essential to ensure optimal therapeutic targeting.

Functionally and developmentally appropriate neurogenesis is a complex process, requiring successful completion of various stages, including neural progenitor cell (NPC) proliferation, neuronal differentiation, migration, and integration (Kempermann et al., 2015). Following experimental TBI in adult rodents, cellular proliferation is upregulated in the subgranular zone (SGZ) of the hippocampal dentate gyrus (DG), resulting in increased numbers of posttrauma-born granule neurons (Kernie et al., 2001; Rola et al., 2006). However, within the altered environment of the traumatized brain, many new hippocampal neurons develop with abnormal dendritic arbors, a phenotype maintained into maturity (Carlson et al., 2014; Villasana et al., 2015; Ibrahim et al., 2016). Trauma also influences migration of immature neurons within the granule cell layer (GCL) and promotes ectopic migration of a small subset of new neurons to the hilar layer of the dentate (HL) (Ibrahim et al., 2016; Robinson et al., 2016; Shapiro, 2017; Littlejohn et al., 2020), suggesting that interventions to improve the fidelity of neurogenic responses after TBI may have utility in guiding appropriate neuronal positioning.

Insulin-like growth factor-1 (IGF1) is an endogenous growth factor that promotes neural plasticity. IGF1 stimulates neurogenesis, process outgrowth, and immature neuron migration in the developing subventricular zone (SVZ) and in hippocampal cell cultures (Hurtado-Chong et al., 2009; Nieto-Estevez et al., 2016). To investigate the efficacy of IGF1 to promote posttraumatic neurogenesis, we have utilized a transgenic mouse with a tet-off construct in which expression of human IGF1 is driven by the GFAP promoter upon the removal of doxycycline-supplemented chow, resulting in postnatal conditional overexpression of IGF1 by astrocytes (Ye et al., 2004). In the controlled cortical impact (CCI) model of contusion TBI, injury triggers a robust wave of reactive astrocytosis in the contused cortex and underlying hippocampus (Dunn-Meynell and Levin, 1997; Myer et al., 2006; Saatman et al., 2006). In this transgenic mouse model, reactive astrocytes upregulate GFAP, thereby driving regional IGF1 overexpression targeted to damaged tissue regions. We have shown a progressive elevation of hippocampal IGF1 levels in astrocyte-specific IGF1 transgenic (IGFtg) mice over the first 3 days after CCI (Madathil et al., 2013). We have further shown that brain-specific, astrocyte-driven IGF1 overexpression stimulates neuronal differentiation, enhances immature neuron dendritic development and improves long-term survival of hippocampal neurons born after contusion TBI (Carlson et al., 2014; Littlejohn et al., 2020). The neurogenic effects of IGF1 were confirmed using a continuous intracerebroventricular infusion of IGF1 to mice after CCI brain injury, resulting in significantly improved recovery of the immature neuron population within the DG (Carlson and Saatman, 2018). Although IGF1 overexpression potentiates injury-induced Akt activity in the hippocampus (Madathil et al., 2013), little is known regarding molecular pathways underlying neurogenic effects of IGF1 in TBI.

The mammalian target of rapamycin (mTOR), a signaling molecule downstream of Akt, is a potent modulator of cell homeostasis and regulates cell growth, energy expenditure, and survival (Lipton and Sahin, 2014). *In vitro* studies have defined a role for mTOR signaling in the regulation of neuronal differentiation, migration, dendritic outgrowth, and survival of newly born neurons (Jaworski et al., 2005; Wahane et al., 2014). mTOR activity is transiently increased within the first day(s) after TBI (Chen et al., 2007; Park et al., 2012; Zhu et al., 2014; Nikolaeva et al., 2016; Wang et al., 2016) and is required for early proliferation of progenitor cells in the SGZ (Wang et al., 2016). However, mTOR activation is postulated to contribute to posttraumatic maladaptive hippocampal plasticity and cognitive impairment associated with posttraumatic epilepsy, a premise supported by improved outcomes associated with administration of the mTOR inhibitor rapamycin (Park et al., 2012; Guo et al., 2013; Butler et al., 2015; Rozas et al., 2015). In light of the potential role for mTOR activity in stimulating aberrant axonal sprouting of granule cell neurons, an outcome that could increase the likelihood of posttraumatic epilepsy, a therapy that drives mTOR activity could be contraindicated for TBI.

To determine whether IGF1 mediates its effects on posttraumatic neurogenesis through mTOR signaling, we subjected mice with astrocyte-specific, conditional IGF1 overexpression to CCI brain injury. We first mapped the patterns, time course, and dependence on injury severity of mTOR activation in the DG, using immunolabeling for phosphorylation of a downstream target, ribosomal protein S6. We then inhibited posttraumatic mTOR activation using a delayed administration of rapamycin, and queried the effects on early hippocampal neurogenesis in wildtype and IGF1 transgenic mice.

## Materials and Methods

### Animals

Heterozygous tTA^GFAP^ mice were bred with heterozygous IGF1^pTRE^ mice as previously described (Ye et al., 2004; Madathil et al., 2013) to generate Tet-off double transgenic mice (tTA^*GFAP*^/IGF1^pTRE^) that express human IGF1 (hIGF1) selectively in astrocytes. IGF1 double transgenic (IGFtg) mice and their wildtype (WT) littermates were fed with doxycycline supplemented mouse chow (200 mg/kg) *ad libitum* until 2 weeks prior to surgery to block hIGF1 transcription. They received standard mouse chow for at least 2 weeks to allow transgene expression prior to surgery/injury. In the absence of doxycycline, hIGF is expressed, but at low levels in uninjured mice due to low basal levels of glial fibrillary acidic protein (GFAP) transcription. In contrast, in mice with CCI, reactive astrocytosis triggers upregulation of GFAP production, resulting in concomitant increases in IGF1 levels in injured regions such as the hippocampus (Madathil et al., 2013).

Mice were provided with food and water *ad libitum* at the University of Kentucky Medical Center animal vivarium where they were housed at a constant temperature (23 ± 2°C) with a 14/10-h light/dark cycle. All procedures involving animals were approved by the University of Kentucky’s Institutional Animal Care and Use Committee, under IACUC protocols 2013–1156 and 2019–3293.

### Controlled Cortical Impact Injury

**FIGURE 1:**
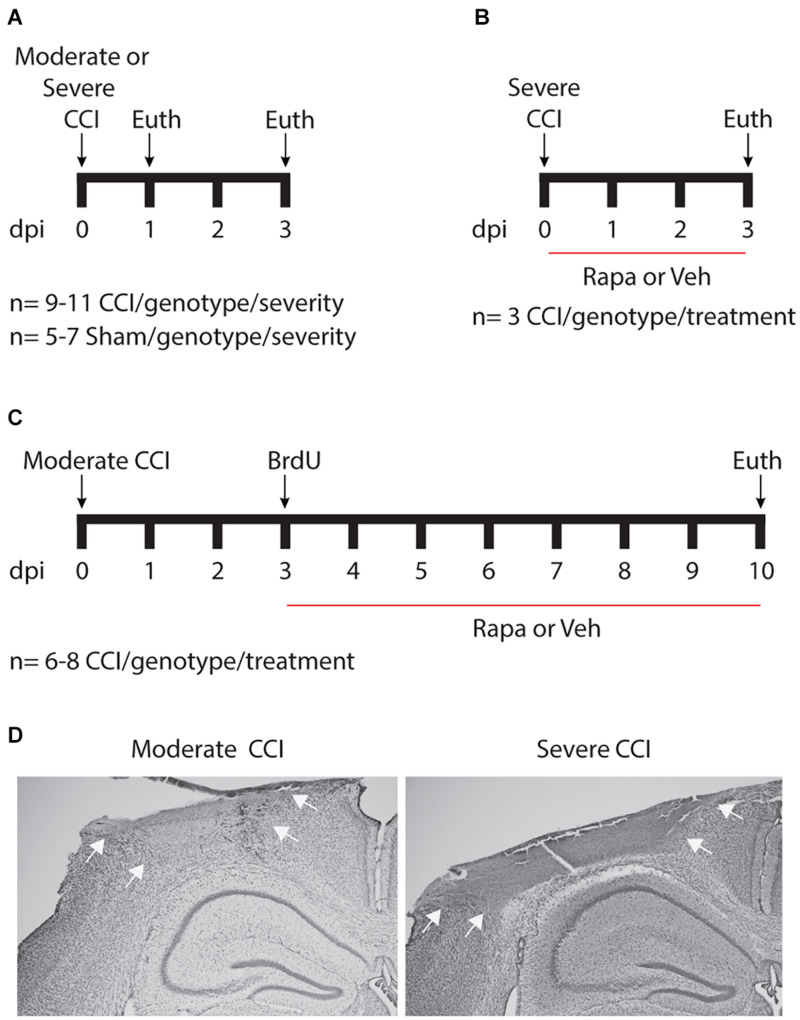
Schematic of experimental design. **(A)** To assess acute posttraumatic mTOR activity, cohorts of mice were euthanized 24 or 72 h after moderate or severe controlled cortical impact (CCI) injury. **(B)** To verify mTOR inhibition by rapamycin (Rapa), brain-injured mice were treated daily with 10 mg/kg Rapa or vehicle (Veh) beginning 1 h after injury for 3 days. **(C)** To examine the contribution of mTOR signaling to posttraumatic neurogenesis, injured mice were treated daily with 10 mg/kg Rapa or Veh beginning on day 3, after mice received three injections of 50 mg/kg BrdU given 4 h apart. dpi, days post-injury. **(D)** Representative images taken from mice with moderate (left) and severe (right) CCI injury. Nissl staining reveals a well-developed cortical contusion at the site of impact, which is larger following severe CCI as compared to moderate CCI. Arrows illustrate the boundaries of the contusion.

Adult (>8 weeks of age) male and female littermates were randomly assigned to CCI or sham injury groups. Surgeons blinded to mouse genotypes and treatments performed moderate or severe CCI on IGFtg (*n* = 59) and WT (*n* = 57) mice. Surgeries were performed as previously described (Madathil et al., 2013). Briefly, anesthesia was induced using 3% isoflurane. After securing the head of the animal in a stereotaxic frame (David Kopf Instruments, CA), anesthesia was maintained using 2.5% isoflurane delivered through a nose cone. A midline scalp incision was made, and a 5 mm diameter craniotomy was performed over the left parietal cortex, lateral to the sagittal suture (2.5 mm lateral) midway between Bregma and Lambda. Cortical contusion was produced using a stereotaxic electromagnetic impactor (Leica Biosystems) with a 3 mm diameter rounded impactor tip, with a velocity of 3.5 m/s to produce moderate (1.0–1.1 mm depth) or severe (1.5 mm depth) brain injury (Figure 1). IGFtg (*n* = 5) and WT (*n* = 7) sham-injured mice received a craniotomy under isoflurane anesthesia. After CCI or sham injury, a circular disk made from dental cement was glued over the craniotomy to protect the brain surface, and the scalp was sutured. Mice were placed on a Hova-Bator Incubator (model 1583, Randall Burkey Co., TX) to maintain body temperature until they regained consciousness, after which they were returned to their home cages. Sham mice for the pS6 immunostaining experiment (Figure 1A) were euthanized 3 days after surgery.

### Rapamycin Administration

Rapamycin (LC Laboratories, Woburn, MA) was dissolved in 100% ethanol (30 mg/ml) and stored at −20°C until use. Immediately before injection, rapamycin was diluted (1:10) into vehicle (5% Tween80, 5% PEG400, 5% EtOH in 1× PBS). Moderately injured IGFtg and WT mice in a 3-day (Figure 1B) and a 10-day (Figure 1C) survival cohort were randomly assigned to either vehicle or rapamycin treatment groups for each experiment. The 3-day survival cohort received daily intraperitoneal (i.p.) injections of 10 mg/kg rapamycin (*n* = 3/genotype) or vehicle (*n* = 3/genotype) beginning 1 h after CCI. The 10-day survival cohort mice received a daily i.p. injection of 10 mg/kg rapamycin (*n* = 8/genotype) or vehicle (*n* = 6–7/genotype) beginning 1 h after the last BrdU injection.

### BrdU Administration

The 10-day survival cohort received three i.p. injections of 50 mg/kg 5-Bromo-2’-deoxyuridine (BrdU, Fisher Scientific, Hampton NH) in saline at 4 h intervals on day 3 after injury (Figure 1C). Intraperitoneal BrdU typically incorporates into dividing cells within 4 h after injection (Taupin, 2007). During handling and disposal of BrdU and all hazardous materials used in this study, proper precautions were taken as approved by the University of Kentucky Office of Environmental Health and Safety and the Institutional Animal Care and Use Committee.

### Immunohistochemistry and Histology

Animals were deeply anesthetized by sodium pentobarbital (Fatal-plus solution, Vortech Pharmaceuticals, Dearborn, MI) and transcardially perfused with heparinized saline followed by 10% buffered formalin. Brains were removed 24 h after post-fixation in 10% formalin, then cryoprotected using 30% sucrose solution and snap frozen in cold isopentanes (≤−25°C). The tissue was cut in a coronal plane at 40 μm thickness. Free-floating immunohistochemistry was performed as previously described on three tissue sections (between −1.5 and −2.9 mm Bregma) selected at 400 μm intervals spanning the injury epicenter (Madathil et al., 2013; Carlson et al., 2014). To increase specific staining of phosphorylated ribosomal protein S6 (pS6), antigen retrieval was performed using 10 mM citric acid pH 6.0 at 60°C. To expose BrdU epitopes, tissue was incubated in 2N HCl (Fisher Scientific) at room temperature with agitation for 1 h, followed by 100 mM borate for 10 min to neutralize residual HCl. The tissue was rinsed overnight in TBS at 4°C. Following pretreatment, sections were washed three times in TBS and then incubated in blocking solution (5% normal horse serum in 0.1% Triton X-100 in TBS) for 1 h. The tissue was incubated with primary antibody at 4°C overnight. Primary antibodies include: pS6 (1:100 rabbit polyclonal anti-pS6, Cat. #2215, Cell Signaling, Boston, MA), a well characterized downstream mTOR effector (Chen et al., 2007); BrdU (1:1000 rat monoclonal anti-BrdU, Cat. #ab6326, Abcam, Boston, MA), a proliferation reporter (Dash et al., 2001); the microtubule protein doublecortin (1:2500 rabbit polyclonal anti-Dcx, Cat. #ab18723, Abcam), a commonly used immature neuron marker (Kempermann et al., 2015); proliferating cell nuclear antigen (1:500 mouse monoclonal anti-PCNA, Cat. #2586, Cell Signaling), a proliferation marker; NeuN (1:500 mouse monoclonal anti-NeuN, Cat. #MAB377, EMD Millipore Corporation, Burlington, MA), a marker for mature neurons; Iba1 (1:1,000 rabbit polyclonal anti-Iba1, Cat. #019-19741, FUJIFILM Wako Pure Chemical Corporation, Richmond, VA), a microglia marker; and glial fibrillary acidic protein (1:1,000 rabbit polyclonal anti-GFAP, Cat. #G9269, EMD Millipore or 1:1,000 chicken polyclonal anti-GFAP, Cat. #GFAP, Aves Labs, Inc., Davis, CA), an astrocyte marker. Secondary antibodies were conjugated with Alexa-488, Cy-3, or Alexa-594 (Invitrogen, Carlsbad, CA). Omission of primary antibody served as a negative control.

### Microscopy Image Analysis

All image acquisition and subsequent analyses were completed by an investigator blinded to the genotype and treatment status of each animal.

#### Positive Immunoreactive Area Imaging and Analysis

For all pS6 analyses, image acquisition was performed using NIS-Elements software (Nikon Instruments, Inc., Melville, NY). After identifying the center of the section, profile images of the DG were taken at 20× magnification (NA 0.75, Nikon, Melville, NY) using a laser scanning confocal microscope equipped with a photomultiplier tube (Nikon Ti Eclipse with C2 plus). All images were captured using identical laser and photodetector settings. Ipsilateral and contralateral hemispheres in each section were imaged, and on a composite image several regions of interest (ROI) were delineated: granule cell layer (GCL), molecular layer (ML), and hilus (HL). Within each ROI, a threshold was set to capture pS6 + cells and a binary mask was applied (see Supplementary Figure 1). The area (mm^2^) of positive pixels was quantified and normalized to the ROI area (mm^2^). Data was averaged from 3 sections per animal.

#### Immature Neuron Image Acquisition and Cell Counts

For all BrdU +, Dcx +, and BrdU + Dcx + cell analyses, the entire ipsilateral DG of the three sections representing the epicenter of injury (Carlson et al., 2014) was imaged on a confocal microscope (Nikon A1R). Images were acquired as a z-stack at 100× magnification under oil (NA 1.45) and at 0.75 μm step intervals through a 20 μm depth to ensure optical resolution to assess the morphology of densely packed immature neurons in the GCL.

To evaluate the effect of IGF1 on numbers of cells that proliferated at 3 dpi, the number of BrdU + and BrdU + Dcx + cells were manually counted in the GCL, HL and the ML of the upper blade of the DG. Cells were determined to reside in the GCL if they were within one cell distance (0–10 μm) of the GCL/HL border (Villasana et al., 2015). Colocalization of markers was confirmed by making 3D reconstructions of the cells of interest to avoid false positives. The volume (mm^3^) of the respective ROI (GCL, ML, and HL) was quantified in each section, for cell counts expressed as volumetric densities. To quantify cell proliferation at 10 dpi, PCNA + cells were manually counted in GCL in one tissue section. PCNA + counts were normalized to the length of the SGZ in the upper and lower blades.

#### Newborn Neuron Localization

Images obtained for BrdU + Dcx + cell counts were used to assess cell localization within the DG. The distance from the hilar border of the GCL (GCL/HL border) to the center of the cell soma was measured for BrdU + Dcx + cells in the upper blade of the GCL. The inner GCL (iGCL) layer is defined as the inner 1/3rd of the GCL area (Kempermann et al., 2003; Mathews et al., 2010; Ibrahim et al., 2016). For quantification, the iGCL and SGZ cell counts (0–50 μm from the HL) were pooled and presented as iGCL. The outer GCL (oGCL) describes the outermost 2/3 of the GCL. Cells localized to the HL, further than 10 μm from the GCL/HL border were considered to be ectopically localized to the HL (Villasana et al., 2015). Cell counts and distances were manually quantified.

### Statistical Analyses

Graphs were generated and data were analyzed using GraphPad Prism software. Data are presented as either box and whisker plots that display the 25th, 50th, and 75th quartiles with minimum and maximum values as whiskers or as mean with error bars depicting standard error of the mean (SEM). Because areas of pS6 immunostaining in the GCL, HL, and ML were equivalent in WT and IGF1 sham controls when compared using *t*-tests, sham data were pooled for analysis. Outliers were identified using Grubbs analysis and are detailed in Supplementary Tables 1, 2. One-way analysis of variance (ANOVA) was used to assess differences among group means except as noted below. For one-way ANOVAs, data sets were evaluated using GraphPad Prism to determine whether standard deviations (SDs) were significantly different among groups. If SDs were equivalent, then a standard ANOVA with Sidak’s *post hoc t*-tests was performed. If SDs were significantly different as determined using the Brown-Forsythe test, then a Welch’s ANOVA was performed and Dunnett’s T3 multiple comparisons tests were used for pairwise comparisons. For analysis of experiments involving rapamycin, planned *post hoc* comparisons were limited to four: WT Veh vs. IGFtg Veh, WT Rapa vs. IGFtg Rapa, WT Veh vs. WT Rapa, and IGFtg Veh vs. IGFtg Rapa. Two-way ANOVA was used to assess cell localization within the GCL (Supplementary Figure 4). Two-way ANOVAs were followed by Sidak’s multiple comparisons tests only when main effect interactions were found to be significant. Details of ANOVA and *post hoc* testing results are provided in tabular format. For all comparisons *p* < 0.05 was considered statistically significant.

## Results

### IGF1 Overexpression Potentiates Injury-Induced mTOR Activation in Predominantly Neuronal Sub-regions of the DG

**FIGURE 2:**
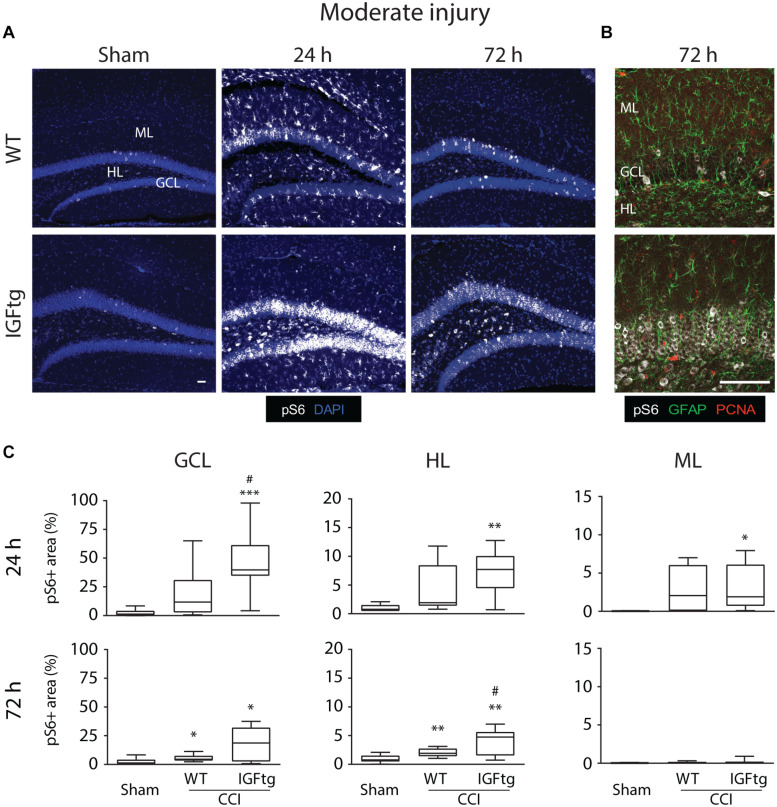
IGF1 potentiates mTOR activity in the dentate gyrus induced by moderate brain injury. **(A)** Representative microphotographs from confocal imaging of immunoreactivity of phospho-S6 Ribosomal Protein (pS6, white) in wildtype (WT) and insulin-like growth factor 1 transgenic (IGFtg) mice following sham injury and 24 and 72 h after moderate controlled cortical impact (CCI) injury. DAPI staining is shown as blue. Scale bar represents 100 μm. **(B)** Confocal images illustrate activation of S6 predominantly in neurons at 72 h after moderate CCI. Astrocytes (GFAP, green) did not colabel with pS6 (white) and most were not proliferating (PCNA, red). Scale bar represents 100 μm. **(C)** The area of pS6 immunostaining was significantly increased in injured IGFtg mice when compared to either injured WT mice or Sham controls in the granule cell layer (GCL) at 24 h and in the hilar layer (HL) at 72 h. In the molecular layer (ML), injury resulted in a small, transient increase in pS6 area at 24 h, which subsided by 72 h post-injury. pS6 + area is presented as a percent of the region of interest area. Data are presented as quartile box plots with min-max. One-way ANOVA, followed by *post hoc* tests: ^∗^*p* < 0.05, ^∗∗^*p* < 0.01, and ^∗∗∗^*p* < 0.001 compared to Sham; ^#^*p* < 0.05 compared to WT CCI. Group sizes: Sham (*n* = 12; 7 WT, 5 IGFtg), 24 h CCI (*n* = 10 WT, 11 IGFtg), 72 h CCI (*n* = 11 WT, 11 IGFtg).

To determine if posttraumatic overexpression of IGF1 amplifies injury-induced mTOR activation in the hippocampal DG, we quantified the total area of cellular immunolabeling for pS6 at 24 and 72 h after moderate or severe CCI (Figure 1A). Because S6 is a downstream target of mTOR activity, phosphorylation of S6 is a widely used indicator of mTOR activation (Lipton and Sahin, 2014; Wang et al., 2016; Millan et al., 2019). In control (uninjured sham) brains of both WT and IGFtg mice, only a few scattered cells exhibit S6 activation (Figure 2A). Following moderate CCI, more widespread S6 activation was noted within the GCL, especially in the IGFtg mice, which also exhibited increased pS6 in the HL. Early S6 activation in the ML was not sustained to 72 h. Colabeling for pS6 with PCNA to detect proliferation and GFAP to detect astrocytes revealed little to no S6 activation within astrocytes at 72 h after moderate injury, despite some evidence of astrocyte proliferation in the ML (Figure 2B). Neuronal S6 activation within the GCL and HL was more pronounced in IGFtg mice and most pS6 + neurons were not labeled with PCNA. Quantification of pS6 staining in WT mice showed a small but statistically significant increase in the GCL and HL at 72 h when compared to sham controls (Figure 2C and Table 1). In contrast, IGF1 overexpressing mice exhibited significantly increased pS6 immunolabeling in all three regions at 24 h when compared to sham controls. This elevation was sustained to 72 h in the GCL and HL of IGFtg mice with moderate CCI. When compared to injured WT mice, IGF1 overexpression stimulated a significantly greater pS6 response in the GCL and HL (Figure 2C and Table 1).

**TABLE 1:** Statistical analysis of regional pS6 immunolabeling data from the ipsilateral hippocampal dentate gyrus.

Moderate injury: Ipsilateral hippocampus				Dunnett’s T3 multiple comparisons test		
Region	Timepoint	*W*-value	*p*-value	Sham vs. WT CCI	Sham vs. IGFtg CCI	WT CCI vs. GFtg CCI
GCL	24 h	19.07	**0.0001**	0.078	***0.0005	#**0.042**
	72 h	8.00	**0.0034**	*0.049	*0.018	0.063
HL	24 h	16.36	**0.0004**	0.078	**0.0014	0.396
	72 h	12.70	**0.0004**	**0.0088	**0.0026	#**0.042**
ML	24 h	9.01	**0.0042**	0.066	*0.020	0.982
	72 h	4.48	**0.033**	0.091	0.234	0.817
**Severe injury: Ipsilateral hippocampus**						
GCL	24 h	9.95	**0.0024**	*0.049	*0.013	0.105
	72 h	5.85	**0.013**	0.519	*0.022	#**0.041**
HL	24 h	15.25	**0.0005**	0.069	**0.0015	0.675
	72 h	24.35	** < 0.0001**	**0.0059	***0.0008	#**0.023**
ML	24 h	11.60	**0.0025**	*0.049	*0.015	0.264
	72 h	33.15	** < 0.0001**	***0.0006	**0.0018	0.244

**FIGURE 3:**
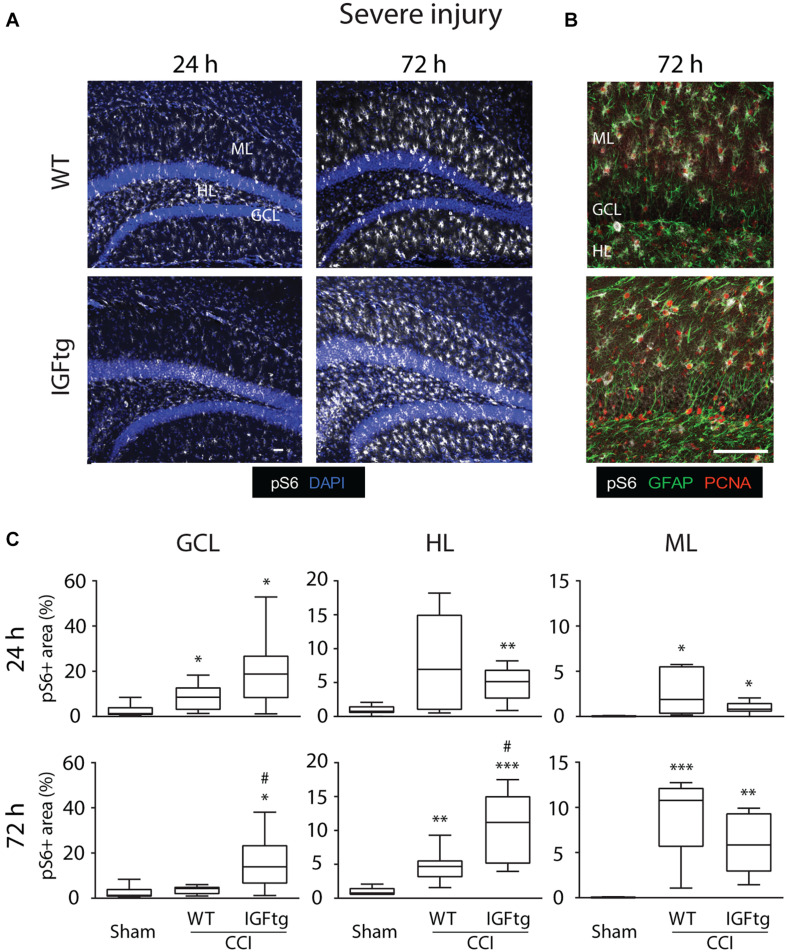
IGF1 selectively enhances granule cell and hilar layer mTOR activity, without affecting delayed S6 activation in proliferating astrocytic in the molecular layer after severe injury. **(A)** Representative confocal images of immunoreactivity of phospho-S6 Ribosomal Protein (pS6, white) in wildtype (WT) and insulin-like growth factor 1 transgenic (IGFtg) mice following sham injury and 24 and 72 h after severe controlled cortical impact (CCI) injury. DAPI staining is shown as blue. Scale bar represents 100 μm. **(B)** Confocal images illustrating colocalization of pS6 (white) and proliferating cell nuclear antigen (PCNA, red) within astrocytes (GFAP, green) of the molecular layer (ML) in the hippocampus ipsilateral to impact at 72 h after severe CCI. Scale bar represents 100 μm. **(C)** In the granule cell layer (GCL) and the hilar layer (HL), IGFtg mice exhibited a significantly larger area of pS6 staining when compared to either injured WT mice or sham controls at 72 h after CCI. In the ML, both WT and IGFtg mice exhibited significant increases in pS6 levels at 72 h. pS6 + area is presented as a percent of the region of interest area. Data are presented as quartile box plots with min-max. One-way ANOVA, followed by *post hoc* tests: ^∗^*p* < 0.05, ^∗∗^*p* < 0.01, and ^∗∗∗^*p* < 0.001 compared to Sham; ^#^*p* < 0.05 compared to WT CCI. Group sizes: Sham (*n* = 12; 7 WT, 5 IGFtg), 24 h CCI (*n* = 9 WT, 10 IGFtg), 72 h CCI (*n* = 9 WT, 10 IGFtg).

*Welch’s one-way analysis of variance (ANOVA, df = 2) was used to compare Sham-injured and controlled cortical impact (CCI) injured wildtype (WT) and insulin-like growth factor-1 transgenic (IGFtg) mice. Analysis for moderate CCI (top) corresponds to Figure 2C and severe CCI (bottom) corresponds to Figure 3C. The regions of interest included the granule cell layer (GCL), hilar layer (HL), and molecular layer (ML), examined at 24 and 72 h after injury. Where appropriate, ANOVA was followed by Dunnett’s T3 multiple comparisons test. Significant group effects and post hoc comparisons are noted in bold. ^∗^Designates comparison of CCI to sham. ^#^Designates a genotype effect. Number of symbols indicates level of significance, as in Figures 2C and 3C.*

Following a severe CCI, S6 activation was increased in the GCL and HL, particularly within the IGFtg mice at 72 h (Figure 3A). Compared to moderate CCI, severe injury initiated a notably more robust involvement of the ML at 72 h in both WT and IGFtg mice, with pS6 + cells exhibiting a glial morphology (Figure 3A). Colabeling with GFAP and PCNA demonstrated that S6 activation in the ML occurred within proliferating astrocytes in both genotypes (Figure 3B). In WT mice, severe injury stimulated a significant increase in S6 activation in the GCL at 24 h, in the HL at 72 h and in the ML at both time points (Figure 3C and Table 1). In contrast, severely injured IGFtg mice showed significant increases in the extent of S6 activation in all three regions at both 24 and 72 h (Figure 3C and Table 1), when compared to shams. As with moderate CCI, S6 activation following severe injury was augmented in IGFtg mice compared to WT mice in the GCL, a predominantly neuronal region, as well as in the HL, but not in the ML (Figure 3C and Table 1).

Interestingly, hippocampal mTOR activation after moderate and severe CCI was bilateral in injured IGFtg mice, while WT mice showed only minor S6 activation in the contralateral HL with severe CCI (Supplementary Figure 2A). Injured mice with IGF1 overexpression exhibited a greater area of pS6 immunolabeling within the GCL and the HL of the hippocampus contralateral to the impact than did sham-injured controls, although this response was more delayed following severe CCI (Supplementary Figure 2B and Supplementary Table 3). The delayed mTOR signaling within astrocytes of the ML so prominent in the ipsilateral hippocampus after severe CCI in both genotypes was absent in the contralateral hippocampal ML.

### Delayed Administration of Rapamycin Modulates Numbers of Proliferated (BrdU+) Cells in IGFtg Mice but Not in Wildtype Mice

We selected a dose of 10 mg/kg rapamycin based on published work related to hippocampal neurogenesis in the mouse CCI model (Butler et al., 2015; Wang et al., 2016). To validate that this dose effectively inhibited mTOR activity in our model, injured WT and IGFtg mice were treated with daily intraperitoneal injections of rapamycin or its vehicle for 3 days beginning 1 h after CCI (Figure 1B). At 72 h after severe CCI, pS6 immunolabeling within the DG ipsilateral to impact, clearly evident in vehicle-treated mice, was effectively silenced by rapamycin treatment (Supplementary Figure 3).

Based on our findings that moderate CCI brain injury in IGFtg mice resulted in IGF1-stimulated mTOR activation more selectively in the GCL without the marked astrocytic S6 activation that accompanied severe CCI, we chose moderate CCI for studies interrogating the role of mTOR signaling in the enhancement of hippocampal neurogenesis by IGF1. Our previous work suggests that IGF1 increases posttraumatic neurogenesis by promoting neuronal differentiation of proliferating NPCs and supporting dendritic development of immature neurons born after TBI rather than by increasing proliferation (Carlson et al., 2014; Carlson and Saatman, 2018; Littlejohn et al., 2020). Therefore, to avoid rapamycin-induced inhibition of cellular proliferation (Paliouras et al., 2012; Wang et al., 2016) which would confound interpretation of neuronal differentiation and immature neuron development, we delayed rapamycin treatment until after the primary wave of proliferation. Three days after CCI, at the peak of trauma-induced proliferation (Dash et al., 2001; Rola et al., 2006), dividing cells in WT and IGFtg mice were labeled with BrdU over an 8 h period, after which rapamycin treatment commenced (Figure 1C).

**FIGURE 4:**
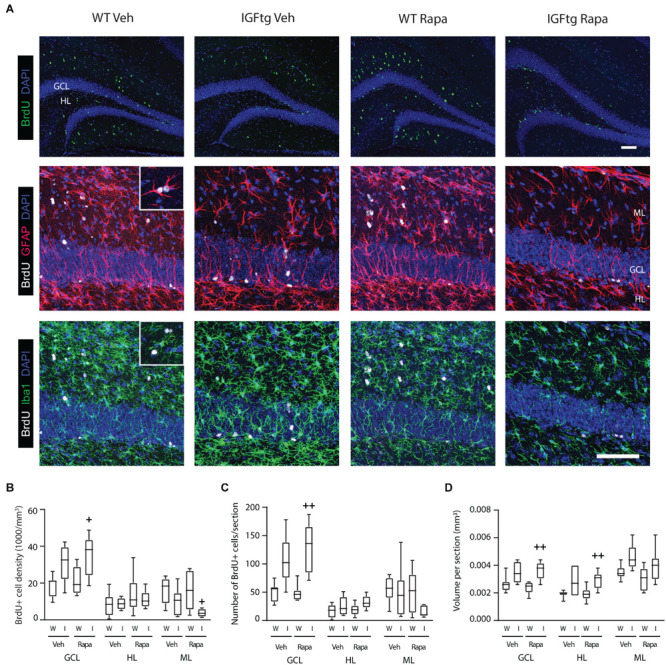
IGF1 overexpression in combination with mTOR inhibition increases the density of proliferated cells in the granule cell layer while decreasing proliferation in the molecular layer. **(A)** *Top row:* Traumatic brain injury results in cellular proliferation (bromodeoxyuridine; BrdU, green) throughout the ipsilateral dentate gyrus of wildtype (WT, W) and IGF1 transgenic (IGFtg, I) mice that received vehicle (Veh) or rapamycin (Rapa). *Middle, bottom rows:* IGFtg mice had more proliferated cells in the granule cell layer (GCL) but fewer within the molecular layer (ML) when compared to WT mice. Colabeling of BrdU (white) with GFAP (red) or Iba1 (green) demonstrated that cells dividing at 3 dpi more frequently phenotyped as astrocytes than microglia in the ML (see insets). DAPI staining is shown as blue. Scale bar represents 100 μm. **(B)** At 10 days after moderate injury, proliferated cells (BrdU +) in the GCL, ML and hilar layer (HL) were quantified. In IGFtg mice treated with Rapa the density (cell number/volume) of BrdU + cells in the GCL was increased while it was decreased in the ML. **(C)** Analysis of non-normalized cell counts revealed increased numbers of BrdU + cells in the GCL of Rapa-treated IGFtg mice. **(D)** GCL and HL volumes were significantly greater in IGFtg mice receiving rapamycin than in similarly treated WT mice. Data are represented as quartile box plots with min-max. One-way ANOVA, followed by *post hoc* tests: + *p* < 0.05 and + + *p* < 0.01 compared to WT Rapa. Group sizes: WT Veh *n* = 7, IGFtg Veh *n* = 6, WT Rapa *n* = 8, IGFtg Rapa *n* = 8.

At 10 dpi, BrdU + cells were observed clustered within the SGZ and distributed throughout the HL and ML (Figure 4A). Proliferated cells within the ML were often colabeled with the astrocyte marker GFAP, whereas far fewer phenotyped as proliferating microglia despite robust labeling with Iba1 indicative of trauma-induced microglial activation (Figure 4A). No appreciable colocalization of BrdU with the mature neuron marker NeuN was observed (data not shown). This was not unexpected as the BrdU + cells here were only 7 days old and NeuN expression begins after 1–2 weeks (Duan et al., 2008; Kernie and Parent, 2010). BrdU + cells were counted separately in the GCL, HL and the upper blade of the ML, and counts were normalized to the region volume (per section) to control for any differences in size across tissue sections or animals. In WT mice, neither the density (Figure 4B) nor the number (Figure 4C) of BrdU + cells was affected in any region by rapamycin treatment. These data suggest that incorporation of BrdU on day 3 and survival of proliferated cells to 10 dpi were not notably altered by mTOR inhibition initiated after BrdU administration. Rapamycin treatment in brain-injured IGFtg mice resulted in a significant increase in both the density and number of proliferated cells in the GCL (Figures 4B,C and Table 2). In contrast, the density of proliferated cells in the ML was notably decreased in the IGFtg mice that received rapamycin (Figures 4A,B). Examination of the relative densities of BrdU + cells in these two regions, the GCL and ML, showed that the mean density of BrdU + cells in the GCL was 1.2 times that in the ML in vehicle-treated WT mice, while the GCL:ML ratio was 3.1 in IGFtg mice. In rapamycin-treated mice, IGF1 overexpression resulted in a sevenfold increase in the GCL density: ML density ratio, from 1.4 in WT mice to 9.5 in IGFtg mice. Proliferation within the HL was not altered by IGF1 overexpression or inhibition of mTOR signaling.

**TABLE 2:** Statistical analysis of posttraumatic proliferation, neuronal differentiation, and positioning in the dentate gyrus.

Neurogenesis measures				*Post hoc* comparisons			
Measure	Figure	*F/W*-value	*p*-value	WT vs. IGFtg (Veh)	WT vs. IGFtg (Rapa)	Veh vs. Rapa (WT)	Veh vs. Rapa (IGFtg)
BrdU + density (GCL)	4B	5.85	**0.0038**	0.083	**+0.020**	0.967	0.798
BrdU + density (HL)	4B	0.93	0.442	–	–	–	–
BrdU + density (ML)	4B	9.02	**0.0028**	0.495	+**0.046**	1.000	0.288
BrdU + cell count (GCL)	4C	10.45	**0.0011**	0.076	++**0.0026**	1.000	0.788
BrdU + cell count (HL)	4C	2.31	0.103	–	–	–	–
BrdU + cell count (ML)	4C	2.33	0.100	–	–	–	–
GCL volume	4D	8.35	**0.0005**	0.098	++**0.007**	0.785	0.947
HL volume	4D	6.70	**0.0063**	0.244	++**0.0096**	1.000	0.998
ML volume	4D	4.00	**0.019**	0.109	0.155	0.854	0.629
BrdU + Dcx + density (GCL)	5B	9.93	**0.0010**	##**0.0067**	+**0.015**	0.808	0.062
BrdU + Dcx + : BrdU (GCL)	5C	8.59	**0.0005**	0.735	+++**0.0006**	1.000	^®^ textbf0.021
iGCL: GCL (BrdU + Dcx +)	5D	3.20	**0.042**	#**0.047**	0.500	0.920	0.910
HL: DG (BrdU + Dcx +)	5E	1.79	0.204	–	–	–	–
PCNA + density (GCL)	6B	1.12	0.360	–	–	–	–

*One-way analysis of variance (ANOVA, df = 3) was used to compare vehicle (Veh) and rapamycin (Rapa) treated brain-injured wildtype (WT) and insulin-like growth factor-1 transgenic (IGF tg) mice. Where appropriate, ANOVA was followed by selected Sidak’s post hoc or Dunnett’s T3 multiple comparisons tests. Significant group effects and post hoc comparisons are noted in bold. ^#^Designates a genotype effect within the vehicle-treated groups. ^+^Designates a genotype effect within the Rapa-treated groups; and ^®^ designates a Rapa treatment effect within a given genotype. BrdU = 5-bromo-2’-deoxyuridine, Dcx, doublecortin; DG, dentate gyrus; GCL, granule cell layer; iGCL, inner GCL; HL, hilar layer; ML, molecular layer; and PCNA, proliferating cell nuclear antigen.*

### IGF1-Mediated Hippocampal Neurogenesis Is Not Diminished by Rapamycin Administration

**FIGURE 5:**
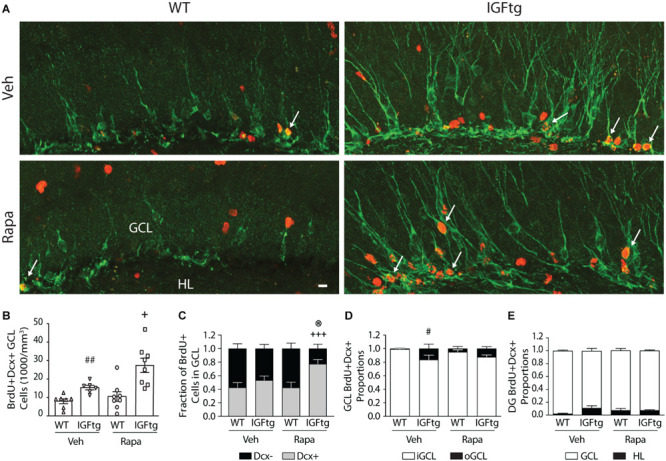
The density and outward migration of posttrauma-born granule neurons is increased in IGF1 overexpressing mice. **(A)** Representative images of immunofluorescence colocalization of doublecortin (Dcx, green), a marker for immature neurons, and the proliferation marker BrdU (red) in the granule cell layer (GCL) of vehicle (Veh) and rapamycin (Rapa) treated wildtype (WT) and IGF1 transgenic (IGFtg) mice at 10 days after injury. White arrows mark double labeled cells. Scale bar represents 20 μm. **(B)** The density of BrdU + Dcx + neurons in the GCL was significantly greater in IGFtg mice compared to their treatment-matched WT groups. **(C)** Rapamycin treatment of IGFtg mice injected with BrdU at 3 dpi resulted in a significantly higher proportion of proliferated cells that differentiated into neurons by 10 dpi. **(D)** The proportion of immature neurons localized to the outer two-thirds of the GCL (oGCL) was increased in vehicle-treated IGFtg mice when compared to WT mice. Inhibition of mTOR activity did not alter migration within the GCL. **(E)** The proportion of newly born dentate gyrus neurons localized to the hilus (HL) at 10 days after moderate injury was not altered by IGF1 overexpression or Rapa treatment. Data are presented as mean + SEM, with individual data points in (B). In Figures C-E, proportions per region are stacked for reference. One-way ANOVA, followed by Sidak’s *post hoc* tests: ^#^*p* < 0.05 and ^##^*p* < 0.01 compared to WT Veh; ^+^*p* < 0.05 and ^+++^*p* < 0.001 compared to WT Rapa; ^®^*p* < 0.05 compared to IGFtg Veh. Group sizes: WT Veh *n* = 7, IGFtg Veh *n* = 6, WT Rapa *n* = 8, IGFtg Rapa *n* = 8.

**FIGURE 6:**
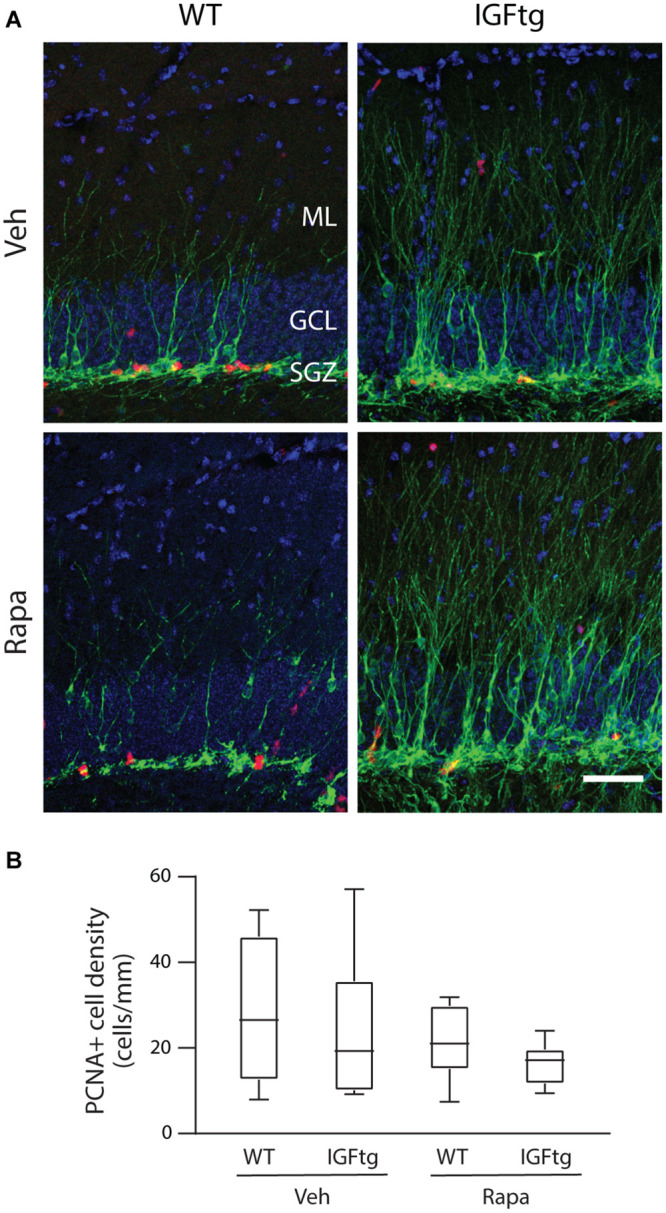
Cellular proliferation at 10 days after injury is not altered by IGF1 overexpression or administration of rapamycin. **(A)** Representative images of doublecortin (Dcx, green) and proliferating cell nuclear antigen (PCNA, red) immunolabeling of the dentate gyrus in wildtype (WT) and IGF1 transgenic (IGFtg) mice that received vehicle (Veh) or rapamycin (Rapa). Inhibition of mTOR signaling does not notably diminish proliferation in the SGZ at 10 dpi for either genotype. DAPI staining is shown as blue. Scale bar represents 100 μm. **(B)** Counts of PCNA + cells within the granule cell layer (GCL), normalized to the length of the SGZ in each tissue section, were equivalent across all groups. Data are represented as quartile box plots with min-max. One-way ANOVA, *p* = n.s. Group sizes: WT Veh *n* = 7, IGFtg Veh *n* = 6, WT Rapa *n* = 8, IGFtg Rapa *n* = 8.

To determine whether mTOR signaling is important for IGF1-mediated cell fate determination of posttrauma-proliferated progenitors, we identified cells labeled with BrdU at 3 days after TBI (prior to the onset of rapamycin treatment) that phenotyped as immature neurons (Dcx +) at 10 dpi (Figure 5A). IGF1 overexpression was associated with a significant, nearly twofold, increase in the density of BrdU + immature neurons in the GCL of vehicle-treated mice (Figure 5B and Table 2). Inhibition of mTOR signaling did not prevent stimulation of neurogenesis by IGF1, but appeared to augment the number of posttrauma-proliferated cells within the GCL that committed to a neuronal fate in IGFtg mice, although this increase did not reach statistical significance (*p* = 0.062, Figure 5B). Because rapamycin treatment in IGFtg mice resulted in higher numbers of BrdU + cells present in the GCL at 10 dpi, we also examined the numbers of newly born neurons as a proportion of all BrdU-labeled cells. Only approximately 40% of cells proliferating at 3 dpi phenotyped as immature GCL neurons at 10 dpi in WT mice, regardless of treatment, whereas rapamycin treatment significantly increased this proportion to nearly 80% in IGFtg mice (Figure 5C and Table 2). These data demonstrate that IGF1 enhances acute posttraumatic neurogenesis and that delayed rapamycin treatment in combination with IGF1 overexpression increases the proportion of proliferated cells that commit to a neuronal phenotype after TBI. In addition to supporting an increase in the density of immature neurons after TBI, IGF1 overexpression was associated with improved dendritic architecture within Dcx + cells (Figures 5A, 6A), replicating our previous work establishing a significant enhancement of immature neuron dendrite length and complexity in mice with IGF1 overexpression (Carlson et al., 2014). Rapamycin treatment appeared to inhibit dendrite development in brain-injured WT mice; however, this inhibitory effect was not notable in IGFtg mice (Figures 5A, 6A).

### Positioning of Posttrauma-Born Immature GCL Neurons Is Not Altered by Rapamycin

Brain injury has been reported to increase outward migration of immature neurons from the SGZ (Villasana et al., 2015; Ibrahim et al., 2016; Shapiro, 2017). Regulation of the positioning of adult-born neurons in the uninjured brain may involve endogenous IGF1 and mTOR signaling pathways (Zhu et al., 2012; Nieto-Estevez et al., 2016), and we have shown that IGF1 overexpression supports greater outward migration of posttrauma-born neurons in the GCL while reducing the proportion mislocalized to the HL (Littlejohn et al., 2020). Examination of the location of new neurons within the GCL revealed, as expected, that the vast majority of 7-day old neurons were found in the SGZ and iGCL for all groups, with 95% or greater in WT mice (Figure 5D). IGF1 overexpression stimulated a small but significant increase in radial migration into the oGCL at 10 dpi (Table 2). Rapamycin did not affect immature neuron migration in either WT or IGFtg injured mice. Although the proportion of newly born GCL neurons within the iGCL did not change with rapamycin treatment (Figure 5D), mTOR inhibition in IGFtg mice resulted in significantly greater numbers of BrdU + Dcx + cells within the first 20 microns from the SGZ/HL border as compared to IGFtg mice receiving vehicle and within the first 40 microns when compared to rapamycin-treated WT mice (Supplementary Figure 4). Only a very small number of 7-day old posttrauma-born immature neurons localized to the HL following moderate CCI, and the proportion of ectopically localized new neurons was not different across groups (Figure 5E).

### Daily Rapamycin Administration Does Not Impair GCL Proliferation at 10 dpi

To determine whether daily administration of rapamycin resulted in suppression of SGZ proliferation in the subacute period after CCI, brain sections were immunolabeled for Dcx and PCNA. In stark contrast to the substantial wave of proliferation in the ML of WT mice captured by BrdU administration at 3 dpi (see Figure 4), at 10 dpi proliferating cells were localized primarily within the SGZ, with few PCNA + cells in the ML (Figure 6A). The density of proliferating cells within the GCL was not significantly altered by either IGF1 overexpression or rapamycin administration (Figure 6B and Table 2).

## Discussion

This study aimed to determine if pro-neurogenic effects of IGF1 in the injured brain are dependent upon mTOR activation. By mapping S6 activation in the hippocampal DG through immunolabeling, we show that conditional, astrocyte-specific IGF1 overexpression selectively modulated regional patterns of posttraumatic mTOR signaling, leading to greater activation within the GCL and HL in the first 3 days after injury. Inhibition of mTOR by rapamycin, however, did not abrogate the enhanced neurogenesis observed in IGFtg mice after CCI brain injury. Rather, mTOR inhibition in mice with elevated brain levels of IGF1 resulted in an increased density of BrdU + cells within the GCL and an increase in the proportion of proliferated cells that differentiated into GCL neurons by 10 dpi. These data provide new insights into the mechanisms of IGF1-mediated neurogenesis in the context of TBI.

### mTOR Activation

Activation of the mTOR pathway has been established in a variety of rodent models of TBI. Rapid increases in hippocampal pS6 levels, assessed by Western blotting, peak between 30 min to 1 day after injury, before returning to baseline by 3 days (Park et al., 2012; Gibb et al., 2015; Wang et al., 2016) or as late as 2 weeks after CCI in mice (Guo et al., 2013). Activation of mTOR signaling in the DG is first observed in neurons, while reactive astrocytes and microglia within the ML contribute to increased pS6 labeling in a delayed manner, at 24–72 h (Chen et al., 2007; Zhu et al., 2014; Gibb et al., 2015; Nikolaeva et al., 2016; Wang et al., 2016). Our data in WT mice is consistent with these previous studies, and is the first study to our knowledge to directly compare hippocampal mTOR activation patterns as a function of injury severity and to implicate mTOR signaling in posttraumatic astrocyte proliferation in the hippocampus. Moderate CCI resulted in only a small degree of S6 activation across the DG of WT mice, while severe CCI produced a progressive increase in ML mTOR signaling which involved proliferating astrocytes. Inhibition of mTOR initiated within the first few hours after TBI has been shown to be neuroprotective and to reduce reactive astrocytosis and microgliosis (Erlich et al., 2007; Guo et al., 2013; Ding et al., 2015; Song et al., 2015; Nikolaeva et al., 2016). Our findings raise the possibility that acute rapamycin administration may reduce reactive gliosis, in part, through suppression of glial proliferation. Indeed, rapamycin has been shown to effectively reduce astrocyte proliferation in the context of hypoxic/ischemic injury and spinal cord injury (Goldshmit et al., 2015; He et al., 2019). However, further studies are warranted to elucidate the specific functions of neuronal and glial mTOR signaling in TBI. It is likely that we did not capture maximal neuronal S6 activation in WT mice, which may occur prior to 24 h postinjury. The current study focused on 24 and 72 h postinjury because during this period hippocampal IGF1 levels are increasing after CCI in this IGFtg mouse model (Madathil et al., 2013) and SGZ proliferation is at its peak (Dash et al., 2001; Rola et al., 2006; Bye et al., 2011).

Overexpression of IGF1 resulted in a more pronounced and widespread activation of S6 in the hippocampus of mice with CCI. The extent of pS6 immunostaining in both the GCL and HL of brain-injured IGFtg mice was significantly increased compared to either sham controls or injured WT mice. IGF1-enhanced mTOR activation in the GCL and HL was evident at both time points after moderate and severe CCI, and was detected bilaterally. This pattern is in clear contrast with S6 activation within the ML, which occurred in a delayed fashion following severe CCI, was largely restricted to the ipsilateral hippocampus, was predominantly localized to proliferating astrocytes, and was not augmented in IGFtg mice. These data suggest that astrocyte-specific IGF1 overexpression stimulated a bilateral activation of neuronal mTOR signaling within the GCL and HL after TBI. In the IGFtg model used here, reactive astrocytes, by way of higher GFAP transcription, drive production of human IGF1 (Ye et al., 2004; Madathil et al., 2013). Human IGF1 protein levels were found to be elevated bilaterally in the hippocampus and to increase from 24 to 72 h after CCI; however, levels were significantly higher in the ipsilateral hippocampus, in keeping with greater astrogliosis ipsilateral to the impact (Madathil et al., 2013). These data raise the possibility that even a low level of IGF1 is sufficient to maximally activate S6 in the GCL and HL, as S6 activation in these regions was not notably greater in the ipsilateral than contralateral hippocampus, at 72 h than 24 h, or with severe compared to moderate CCI. It is worth noting that in both WT and IGFtg mice, pS6 immunolabeled cells were located throughout the inner and outer layers of the GCL following injury, as noted by others (Chen et al., 2007; Nikolaeva et al., 2016). Preferential pS6 staining within SGZ NPCs reported at 24 h after CCI is transient and was not observed at 4 or 48 h (Wang et al., 2016). It is possible that we did not detect this transient activation due to slight differences in the injury model across labs contributing to a shift in the timing of S6 activation in SGZ NPCs.

### Early Proliferation and Acute Survival

Brain injury stimulates proliferation throughout all layers of the DG, peaking on the order of 2–3 days following injury (Dash et al., 2001; Rola et al., 2006; Bye et al., 2011; Gao and Chen, 2013). While the majority of proliferating cells are glial cells located outside the GCL, proliferation is also increased in the SGZ, resulting in the generation of new granule neurons (Kernie et al., 2001; Sun et al., 2005; Gao and Chen, 2013; Wang et al., 2016). By delaying rapamycin administration until after BrdU incorporation on day 3 after injury, we avoided suppression of early posttraumatic proliferation of NPCs (Wang et al., 2016) and astrocytes (He et al., 2019) by rapamycin, as demonstrated by equivalent BrdU + cell counts in vehicle- and rapamycin-treated WT mice across all subregions of the DG. In mice overexpressing IGF1, rapamycin treatment resulted in more BrdU + cells detected in the GCL and fewer in the ML. IGF1 overexpression alone resulted in a similar trend toward higher BrdU + cell numbers, suggesting a mild effect on either proliferation at 3 dpi or enhanced survival of proliferated cells to 10 dpi within the GCL. IGF1 has been shown to stimulate NPC proliferation *in vitro* as well as *in vivo*, in both uninjured and ischemic rats (Aberg et al., 2003; Dempsey et al., 2003). However, other studies suggest that IGF1 promotes neurogenesis by enhancing neuronal differentiation rather than proliferation (Arsenijevic and Weiss, 1998; McCurdy et al., 2005; Carlson et al., 2014; Littlejohn et al., 2020). It is possible that rapamycin, which has shown to be neuroprotective in TBI, when combined with IGF1, acts to increase survival of proliferated cells within the GCL, leading to a significant increase in GCL BrdU + cell density. Inhibition of mTOR has been shown to reduce astrocyte proliferation (Goldshmit et al., 2015; He et al., 2019), which could contribute to a decrease in ML proliferation in rapamycin-treated mice. However, this regional decline was only noted in the IGFtg mice. As IGF1 is thought to support astrocyte and microglial proliferation (O’Kusky and Ye, 2012), the mechanism underlying decreased proliferation in the ML in IGFtg mice receiving rapamycin requires further investigation.

One caveat to consider in interpreting our BrdU data is that contusion TBI is associated with a transient opening of the BBB throughout the contused cortex, which peaks within an hour in the cortex and diminishes over 24 h postinjury (Bharadwaj et al., 2016). Hippocampal BBB breakdown may also occur at higher levels of injury. Opening of the BBB could influence local BrdU concentrations and cellular uptake in the brain if BrdU is not administered at saturating concentrations (Taupin, 2007). We expect that this potential confound is minimal in our study for several reasons: (1) We compared proliferation across groups of injured mice, not between sham and injured mice; (2) Although 200–300 mg/kg is recommended as a saturating dose based on rat studies (Taupin, 2007), the 50 mg/kg dose we employed has been shown to label on the order of 90% of S-phase proliferating cells in the DG (Burns and Kuan, 2005) and has been used in multiple studies of hippocampal neurogenesis in mice, including those involving TBI (Kempermann et al., 2003; Rola et al., 2006; Shi et al., 2007; Gao et al., 2009; Peters et al., 2018); (3) We used a moderate level of CCI which is associated with minimal hippocampal BBB disruption (Orhan et al., 2016; Yu et al., 2019) and administered BrdU in a delayed fashion (3 dpi). Mice administered 50 mg/kg BrdU 3 times on day 2 after CCI did not show increased BrdU labeling within the GCL when compared to sham controls (Peters et al., 2018).

### Generation of Newborn Neurons

TBI causes a loss of immature hippocampal neurons in the GCL over several days, which is followed by spontaneous recovery over 1–2 weeks, supported by increased proliferation of NPCs in the SGZ (Kernie et al., 2001; Rola et al., 2006; Yu et al., 2008; Carlson et al., 2014). Using the same IGFtg mouse model as in the current study, we previously showed that while astrocyte-driven overexpression of IGF1 did not protect against early TBI-induced loss of immature neurons, it significantly increased the numbers of neurons born within the first week after CCI that phenotyped as immature neurons at 10 days (Carlson et al., 2014) and as mature GCL neurons at 6 weeks (Littlejohn et al., 2020). The ability of IGF1 to improve recovery of the immature neuron population was confirmed using intracerebroventricular infusion of IGF1 in mice with CCI (Carlson and Saatman, 2018). In the current study, we provide independent replication of our previous findings, demonstrating that IGF1 overexpression resulted in an increased density of newborn neurons in the GCL in brain-injured mice.

Numerous lines of evidence support a role for IGF1 signaling in stimulating neuronal differentiation (Arsenijevic and Weiss, 1998; Aberg et al., 2000; McCurdy et al., 2005; Otaegi et al., 2006). Rapamycin treatment attenuates hippocampal NPC differentiation stimulated by both IGF1 and insulin (Han et al., 2008; Zhang et al., 2014), implicating mTOR as a downstream mediator. Indeed, inhibition of mTORC1 activity using an shRNA approach resulted in decreased numbers of newborn SVZ neurons, while excessive activation of mTORC1 resulted in increased neuronal differentiation without an effect on proliferation (Hartman et al., 2013). Therefore, we hypothesized that rapamycin treatment would block IGF1-stimulated increases in newborn neuron numbers after injury. Interestingly, inhibiting mTOR activation did not attenuate the neurogenic potential of IGF1, but rather appeared to further enhance it, with an effect size nearing statistical significance (*p* = 0.06). Rapamycin treatment in IGFtg mice altered differentiation of cells proliferating at 3 dpi, as indicated by an increase in the proportion of BrdU + cells that expressed the immature neuron marker Dcx at 10 dpi. This study provides novel evidence that, in the context of traumatic injury, mTOR provides negative feedback on neuronal differentiation downstream of the IGF1 pathway.

Under conditions of prolonged stimulation of IGF1 or insulin receptors, PI3K and Akt activation result in phosphorylation and eventual degradation of insulin receptor substrate-1 (IRS-1) as a means of negative feedback control (Haruta et al., 2000; Harrington et al., 2005). Akt activation in response to IGF1 receptor binding activates mTORC1, which in turn inhibits Akt, thereby limiting IGF1-mediated actions (O’Reilly et al., 2006; Chen et al., 2010). Rapamycin, by inhibiting mTORC1, releases these negative feedback mechanisms, resulting in increased pAkt and IRS-1 (O’Reilly et al., 2006; Carracedo et al., 2008; Carloni et al., 2010). Our data suggest that posttraumatic rapamycin administration, in IGFtg mice with prolonged elevation of brain IGF1 levels, releases negative feedback on IGF1 signaling, resulting in enhanced neurogenesis through increased proliferation and neuronal differentiation.

In brain-injured WT mice, delayed onset inhibition of mTOR signaling did not affect newborn neuron density in the GCL. To our knowledge, this is the first demonstration that mTOR activation is not required for neuronal differentiation of proliferated NPCs in the context of TBI. Rapamycin treatment initiated within 20–30 min and continuing for several days after CCI in mice has been shown to block TBI-induced increases in immature neuron numbers in the GCL (Butler et al., 2015). This effect, however, likely stems from inhibition of NPC proliferation, given that rapamycin treatment spanning the first 2 days after TBI effectively suppresses posttraumatic NPC proliferation (Wang et al., 2016).

Our studies do not distinguish effects of IGF1 or rapamycin on specific developmental stages of hippocampal NPCs and neuronal progenitor cells. The neurogenic niche of the SGZ contains quiescent, radial-glia-like NPCs which divide asymmetrically, giving rise to amplifying NPCs that then undergo rapid symmetric division to generate neuroblasts (Plumpe et al., 2006; Encinas and Enikolopov, 2008; Kernie and Parent, 2010). Doublecortin expressing neuroblasts represent committed neuronal precursors, but at early stages retain proliferative capacity and exhibit a bipolar morphology, typically oriented horizontally within the SGZ. In contrast, postmitotic, Dcx + neuroblasts, or immature neurons, extend an apical dendrite transversely through the GCL, branching within the ML. Although increased SGZ proliferation in response to TBI is well established, few studies have examined the effects of TBI on specific subsets of NPCs and neuronal precursor cells. Yu et al. (2008) used nestin-GFP transgenic mice to establish that CCI increases proliferation of quiescent NPCs in the SGZ by 3 days, while an increase in proliferation of Dcx + neuronal precursor cells occurs later, at 7 dpi. Using rigorous cell counting methodology, Gao et al. (2009) corroborated that TBI stimulates proliferation of quiescent rather than amplifying NPCs.

### Migration and Dendritic Development

Adult-born, immature neurons position within 1–2 weeks of their birth in the uninjured brain (Kuhn et al., 1996; Kempermann et al., 2003). While during embryonic development hippocampal NPCs give rise to neurons that localize preferentially in the outer third of the GCL, the majority of neurons born within the SGZ of adults migrate only a short distance and reach maturity in the iGCL (Kempermann et al., 2003; Mathews et al., 2010). Traumatic injury to the brain disturbs the normal pattern of adult-born neuron migration, resulting in increased numbers of immature neurons localized within the outer two-thirds of the GCL (Villasana et al., 2015; Ibrahim et al., 2016; Tensaouti et al., 2020). IGF1 overexpression stimulated outward migration in the injured brain, yielding a significantly greater proportion of posttrauma-born immature neurons located in the middle and outer GCL in vehicle-treated IGFtg mice than in WT mice. Alterations in positioning of posttrauma-born GCL neurons are observed even as late as 6 weeks postinjury (Littlejohn et al., 2020), suggesting that new neurons that migrate to the oGCL are not eliminated. Hyperactivation of downstream effectors of IGF1 such as Akt or mTOR/S6 causes acceleration of newborn neuron migration into the outer GCL (Duan et al., 2007; Kim et al., 2009; Zhou et al., 2013). Deletion of PTEN, disinhibiting mTOR signaling, also results in increased migration of newborn granule neurons out of the inner third of the GCL (Getz et al., 2016). Increased outward migration stimulated by Akt or mTOR overactivation can be attenuated in uninjured adult mice by rapamycin treatment (Zhou et al., 2013). Here, rapamycin treatment did not significantly reduce outward migration associated with IGF1 expression, raising the possibility that additional factors such as chemokines (Schultheiss et al., 2013) or changes in guidance-related molecules are involved.

Overactivation of mTOR signaling is posited as a mediator of pathologies associated with epilepsy such as ectopic localization of newborn neurons into the HL, aberrant mossy fiber sprouting, and seizures (Meikle et al., 2007; Pun et al., 2012). Thus, elevating brain levels of IGF1 following TBI, which we show enhances early posttraumatic mTOR signaling and results in increased hippocampal neurogenesis, raises a potential concern regarding the stimulation of ectopic localization of newborn neurons and the formation of aberrant circuits. In animal models of epilepsy, inhibition of mTOR reduces mossy fiber sprouting but does not reduce ectopic hilar neurons (Buckmaster and Lew, 2011; Guo et al., 2013; Hester et al., 2016), suggesting that mislocalization of newborn neurons to the HL may be regulated through other pathways. We found no significant increase in ectopic localization of posttrauma-born neurons at 10 dpi in mice with IGF1 overexpression, and no effect of rapamycin administration. In our earlier work, we show that the small proportion of newborn neurons that mislocalize to the dentate hilus after CCI in WT mice is reduced at 6 weeks postinjury in IGFtg mice (Littlejohn et al., 2020). Nonetheless, further studies are needed to examine the functional circuitry involving newborn neurons generated in the injured brain during periods of IGF1 overexpression or administration.

In addition to increased migration within the GCL, immature neurons exhibit an abnormal morphology after TBI, with a less complex dendritic arbor due to shorter dendrites and less branching (Carlson et al., 2014; Ibrahim et al., 2016; Shahror et al., 2020). In brain-injured WT mice, rapamycin treatment appeared to further impair the dendritic development of immature hippocampal neurons, consistent with a role for mTOR in regulating dendritic arborization of developing neurons during adult neurogenesis (Jaworski et al., 2005; Kim et al., 2009; Skalecka et al., 2016). In contrast, IGF1 is essential for development of neuronal dendritic arbors (Bondy and Cheng, 2004; Skalecka et al., 2016) and has been shown to rescue dendritic arborization deficits associated with developmental disorders (de Souza et al., 2017; Chen et al., 2020). We have shown that dendritic complexity of immature hippocampal neurons is significantly enhanced by IGF1 overexpression (Carlson et al., 2014). This IGF1-mediated improvement in architecture of immature neuron dendrites was observed again in the current study, and was not eliminated by rapamycin treatment.

## Conclusion and Considerations

The function and regulation of adult neurogenesis in the injured or diseased brain is not well understood. Nonetheless, harnessing endogenous neurogenesis for the replacement of damaged or dead neurons or the repair of disrupted circuitry is an attractive therapeutic target for brain injury or neurodegenerative diseases associated with hippocampal damage and deficits in hippocampally mediated behaviors such as cognition. IGF1 supports axon and dendrite growth, and promotes neurogenesis in the aging brain as well as in conditions of injury such as stroke and TBI (Madathil and Saatman, 2015). However, concerns exist regarding stimulating neurogenesis after TBI (Neuberger et al., 2017), especially because increased neurogenesis linked to seizures may underlie aberrant connectivity (Parent et al., 1997; Jessberger et al., 2005). Because IGF1 acts to increase Akt/mTOR signaling and mTOR inhibition is effective in reducing certain hallmarks of epilepsy and posttraumatic epilepsy (Buckmaster and Lew, 2011; Guo et al., 2013; Butler et al., 2015; Jiang et al., 2015; Hester et al., 2016), it is important to determine whether IGF1-based therapies for TBI increase the risk for posttraumatic epilepsy.

We present initial data that argues that the neurogenic effects of IGF1 in TBI do not rely on mTOR signaling. Rather, in the presence of rapamycin, the ability of IGF1 to stimulate proliferation and/or support survival of proliferated cells in the GCL as well as increase generation of new neurons is enhanced. These converging lines of evidence suggest that, under conditions of prolonged IGF1 elevation, mTOR inhibition results in disinhibition of a negative feedback loop in the IGF1 signaling pathway, resulting in greater IGF1-mediated effects on hippocampal neurogenesis. These data raise the possibility that IGF1-based therapies could be used in combination with rapamycin to stimulate functional plasticity while reducing the likelihood for posttraumatic seizures and aberrant mossy fiber sprouting. Although our previous work suggests that IGF1 contributes to improved cognitive function (Saatman et al., 1997; Madathil et al., 2013; Carlson and Saatman, 2018; Littlejohn et al., 2020), additional studies are required to assess the long-term consequences of IGF1-stimulated neurogenesis after TBI.

## Data Availability Statement

The original contributions presented in the study are included in the article/Supplementary Material. Further inquiries can be directed to the corresponding author.

## Ethics Statement

The animal study was reviewed and approved by the University of Kentucky IACUC.

## Author Contributions

EL and KS contributed to the conception, study design, and manuscript drafting, performed data interpretation and statistical analysis. EL, AD, HW, RC, BJ, and KS performed the manuscript revisions. EL, AD, HW, RC, and JJ performed the experiments. EL, AD, BJ, RC, and KS generated the figures. EL was the first author of this work. All authors read and approved the final manuscript.

## Conflict of Interest

The authors declare that the research was conducted in the absence of any commercial or financial relationships that could be construed as a potential conflict of interest.
