# Supplementary material for: IGF1-Stimulated Posttraumatic Hippocampal Remodeling Is Not Dependent on mTOR
Source: Front Cell Dev Biol. 2021 May 20;9:663456. doi: 10.3389/fcell.2021.663456 (PMC8174097; doi:10.3389/fcell.2021.663456)
Supplement: Supplementary file 1 [file Data_Sheet_1.pdf]

Supplementary Material

1 Supplementary Tables

1.1 Supplementary Table 1

Injury Severity	Time	Genotype	Ipsilateral (Figures 2 & 3)			Contralateral (Suppl. Figure 2)		
			GCL	HL	ML	GCL	HL	ML
Sham		WT/IGFtg	0	1	0	0	0	1
Moderate CCI	24 h	WT	0	1	1	0	0	0
	24 h	IGFtg	0	1	0	0	0	1
	72 h	WT	1	1	1	1	0	0
	72 h	IGFtg	0	0	1	0	0	1
Severe CCI	24 h	WT	0	0	0	1	0	1
	24 h	IGFtg	0	0	0	0	0	0
	72 h	WT	1	0	1	1	1	1
	72 h	IGFtg	1	1	0	0	0	1

Identification of Grubbs outliers for pS6 immunolabeling quantification. The Grubbs test was used to identify outliers in groups of wildtype (WT) and insulin-like growth factor-1 transgenic (IGFtg) mice subjected to sham injury, moderate controlled cortical injury (CCI, top) and severe CCI (bottom) for measures of the percent area of phosphorylated S6 ribosomal protein (pS6) immunoreactivity in subregions of the hippocampal dentate gyrus ipsilateral and contralateral to the impact at 24 h and 72 h post-injury. This analysis pertains to data presented in Figures 2, 3 and Supplemental Figure 2, as noted. GCL = granule cell layer, HL = hilar layer, and ML = molecular layer.

1.2 Supplementary Table 2

Figure	Measure	Region	WT Veh	IGFtg Veh	WT Rapa	IGFtg Rapa
4B	BrdU+ cell density	GCL	0	0	1	0
		HL	0	1	0	0
		ML	0	0	0	1
4C	BrdU+ cell counts	GCL	0	0	1	0
		HL	0	1	1	0
		ML	0	0	0	1
4D	Volume (ROI)	GCL	0	0	0	0
		HL	1	0	0	0
		ML	0	0	0	0
5B	BrdU+Dcx+ density	GCL	0	0	0	0
5C	BrdU+Dcx+ : BrdU	GCL	0	0	0	1
5D	iGCL : oGCL (BrdU+Dcx+)	GCL	1	0	1	0
5E	HL : DG (BrdU+Dcx+)	DG	0	0	0	0
6B	PCNA+ cell density	GCL	0	0	0	0

Identification of Grubbs outliers for neurogenesis measures. The Grubbs test was used to identify outliers in the density, number and distribution of proliferating cells (BrdU+) and posttrauma-born immature neurons (BrdU+Dcx+) in vehicle (Veh) and rapamycin (Rapa) treated brain-injured wildtype (WT) and insulin-like growth factor-1 transgenic (IGFtg) mice. This analysis pertains to data in Figures 4, 5 and 6, as noted. BrdU= 5-bromo-2′-deoxyuridine, Dcx = doublecortin, DG = dentate gyrus, GCL = granule cell layer, HL = hilar layer, ML = molecular layer, iGCL = inner granule cell layer, oGCL = outer granule cell layer, and PCNA=proliferating cell nuclear antigen.

1.3 Supplementary Table 3

Moderate Injury: Contralateral Hippocampus — Post-hoc comparisons

Region	Timepoint	F/W value	p value	Sham vs WT CCI	Sham vs IGFtg CCI	WT CCI vs IGFtg CCI
GCL	24 h	14.72	**0.0008**	0.129	** **0.0019**	# **0.023**
	72 h	10.38	**0.0011**	0.842	** **0.0027**	## **0.0031**
HL	24 h	12.47	**0.0015**	0.177	** **0.0031**	0.212
	72 h	8.71	**0.0023**	0.115	** **0.0075**	# **0.020**
ML	24 h	16.38	**0.0006**	* **0.049**	** **0.0025**	## **0.0094**
	72 h	7.02	**0.0034**	0.540	** **0.0028**	0.067

Severe Injury: Contralateral Hippocampus

Region	Timepoint	F/W value	p value	Sham vs WT CCI	Sham vs IGFtg CCI	WT CCI vs IGFtg CCI
GCL	24 h	3.17	**0.069**	0.515	0.105	0.177
	72 h	4.22	**0.034**	0.846	* **0.049**	0.054
HL	24 h	6.74	**0.0088**	* **0.027**	0.093	0.914
	72 h	10.07	**0.0022**	* **0.048**	* **0.013**	# **0.031**
ML	24 h	1.07	0.359	--	--	--
	72 h	2.58	0.096	--	--	--

Statistical analysis of regional pS6 immunolabeling data from the contralateral hippocampal dentate gyrus. One-way analysis of variance (ANOVA, df=2) was used to compare sham-injured and controlled cortical impact (CCI) injured wildtype (WT) and insulin-like growth factor-1 transgenic (IGFtg) mice. Analysis for moderate CCI (top) and severe CCI (bottom) corresponds to Supplemental Figure 1. The regions of interest included the granule cell layer (GCL), hilar layer (HL), and molecular layer (ML), examined at 24 h and 72 h after injury. Where appropriate, ANOVA was followed by Sidak's post-hoc or Dunnett's T3 multiple comparisons tests. Significant group effects and post-hoc comparisons are noted in bold. * designates comparison of CCI to sham; # designates a genotype effect.

2 Supplementary Figures

2.1 Supplementary Figure 1

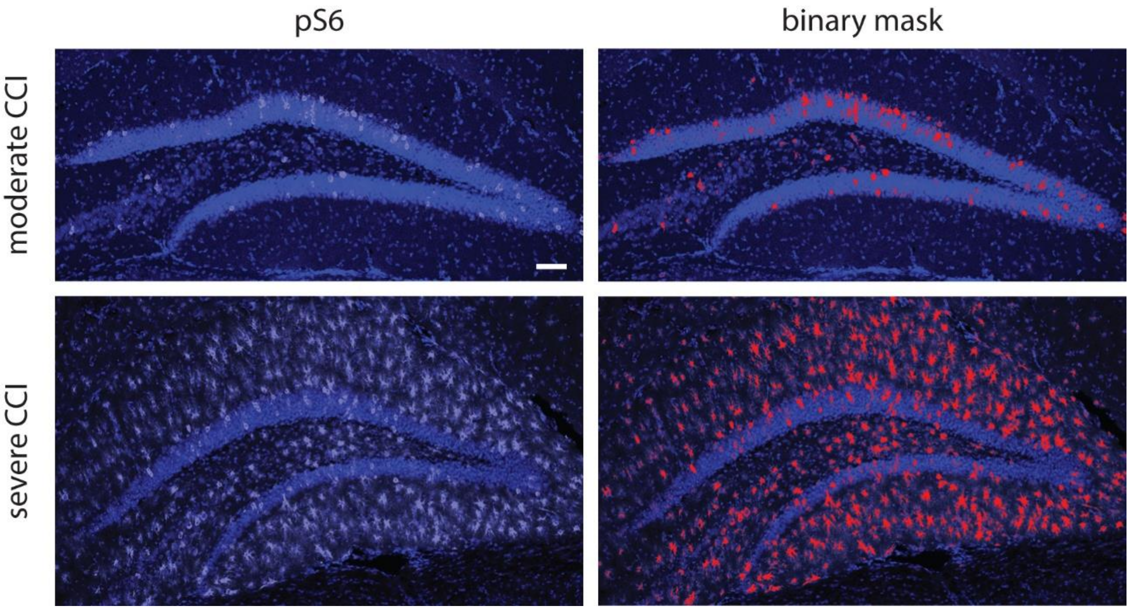

Intensity thresholding to create binary mask for area quantification of pS6 immunofluorescence. Example images of phospho-S6 Ribosomal Protein (pS6, white) immunostaining (left) in the ipsilateral dentate gyrus of IGFtg mice 72 h after moderate (top) or severe (bottom) controlled cortical impact (CCI) and the corresponding binary mask (right) created using intensity thresholding which captures neuronal and glial pS6 labeling. DAPI staining is shown in blue. Scale bar represents 100 µm.

2.2 Supplementary Figure 2

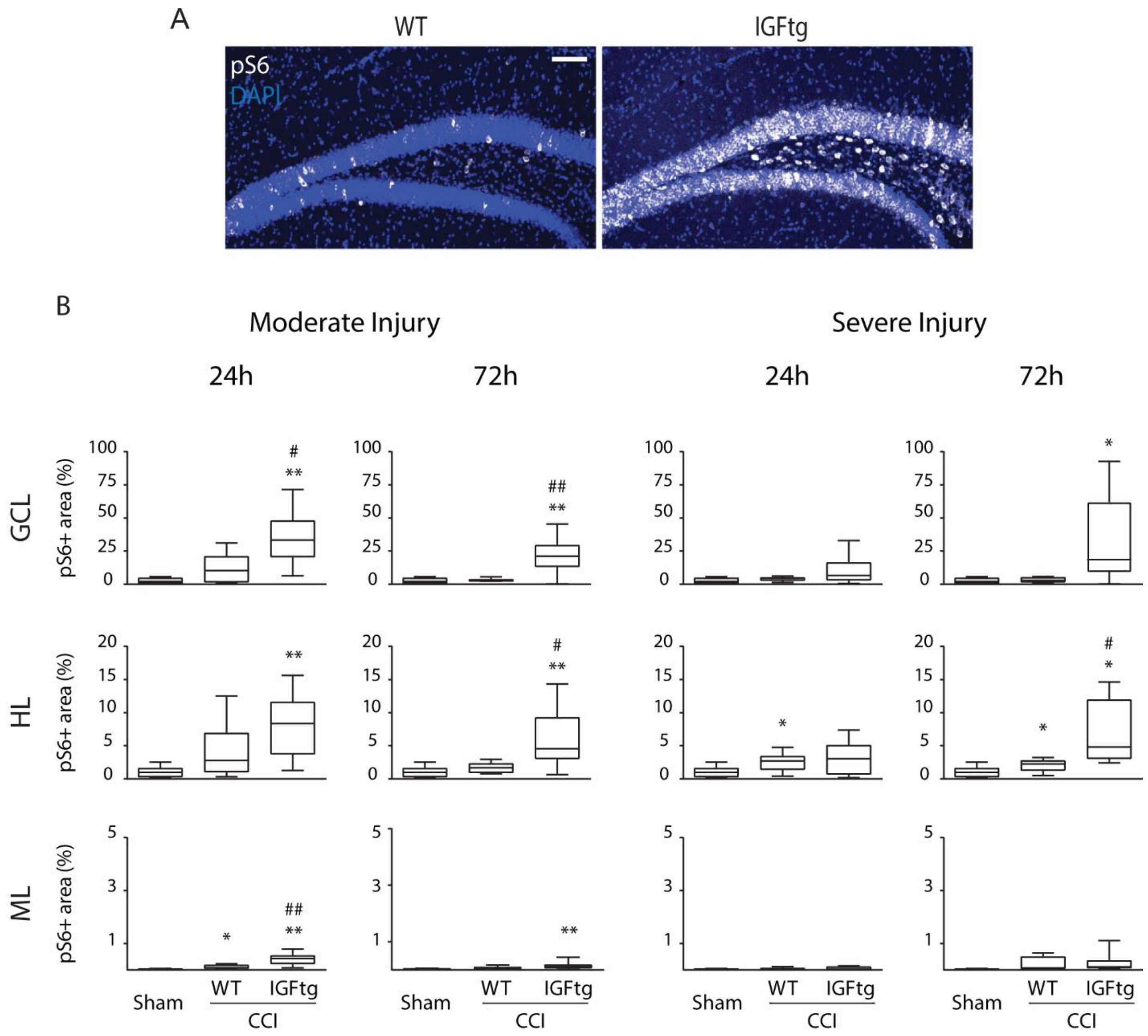

S6 activation is bilateral in the hippocampus in IGF1 overexpressing mice in response to moderate or severe CCI. (**A**) Representative photomicrographs of phospho-S6 Ribosomal Protein (pS6, white) immunoreactivity in the contralateral dentate gyrus (DG) of injured wildtype (WT) and insulin-like growth factor-1 transgenic (IGFtg) mice (72 h after moderate injury shown). DAPI staining is shown in blue. Scale bar represents 100 µm. (**B**) Quantification of the area of pS6 cellular immunolabeling reveals minimal posttraumatic upregulation of mTOR activity in the contralateral hippocampus of WT mice. In contrast, IGFtg mice show robust S6 activation in the contralateral granule cell layer (GCL) at both 24 h and 72 h postinjury, with significant increases in the hilar layer (HL) as well. pS6+ area is presented as a percent of the region of interest area. Data are presented as quartile box plots with min-max. One-way ANOVA, followed by post-hoc tests: * $p<0.05$ and ** $p<0.01$ compared to Sham; # $p<0.05$ and ## $p<0.01$ compared to WT CCI (controlled cortical impact). Group sizes: Sham (n=12; 7 WT, 5 IGFtg); Moderate CCI 24h (n=10 WT, 11 IGFtg) and 72 h (n=11 WT, 11 IGFtg); Severe CCI 24h (n=9 WT, 10 IGFtg) and 72 h (n=9 WT, 10 IGFtg).

2.3 Supplementary Figure 3

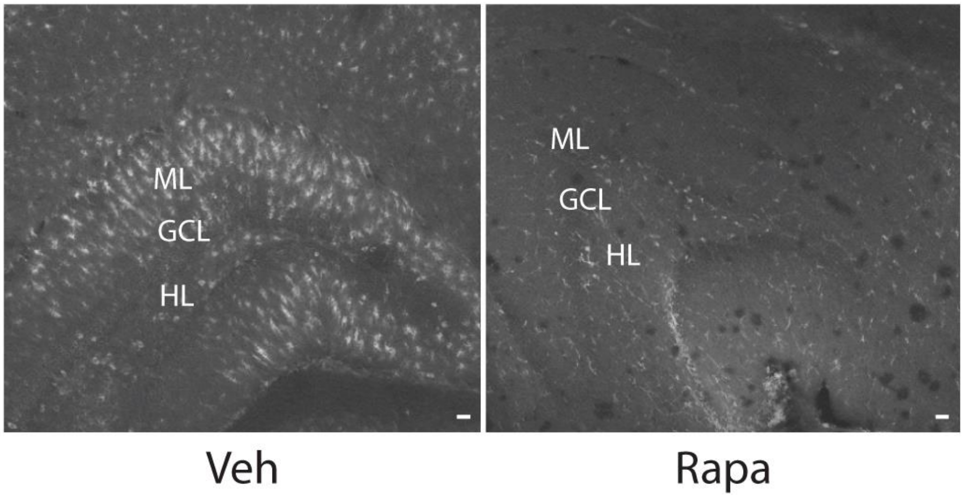

Daily administration of rapamycin effectively inhibits acute posttraumatic mTOR activation. Representative images of phospho-S6 Ribosomal Protein (pS6, white) immunoreactivity in the ipsilateral dentate gyrus following daily injections of vehicle (Veh) and 10 mg/kg rapamycin (Rapa) until euthanasia at 3 days after moderate injury. Scale bar represents 100 µm.

2.4 Supplementary Figure 4

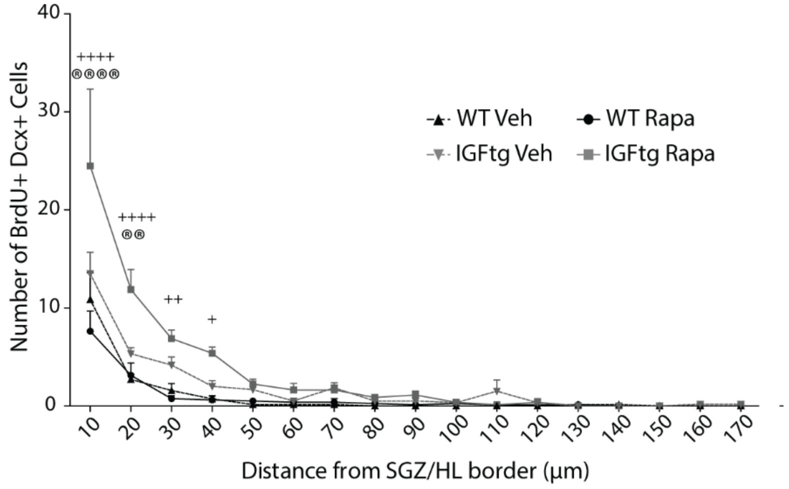

Numbers of newly born neuron within the inner granule cell layer (iGCL) are increased by rapamycin treatment in brain-injured mice with IGF1 overexpression. Quantification of numbers of immature neurons proliferated at 3 days post-injury (dpi) as a function of their distance from the subgranular zone (SGZ)/hilar border assessed at 10 dpi. Rapamycin (Rapa)-treated IGFtg mice have significantly more proliferated immature neurons localized to the iGCL compared to vehicle (Veh)-treated IGFtg mice or to Rapa-treated WT mice. Data presented as mean + SEM. Two-way ANOVA with Sidak's multiple comparisons tests: ®® $p<0.01$ and ®®®® $p<0.0001$ for IGFtg Veh vs. IGFtg Rapa; + $p<0.05$, ++ $p<0.01$ and ++++ $p<0.0001$ for WT Rapa vs. IGFtg Rapa. Group sizes: WT Veh n=7, IGFtg Veh n=6, WT Rapa n=8, IGFtg Rapa n=8.
